# Mass Spectrometry Analysis of Neurotransmitter Shifting during Neurogenesis and Neurodegeneration of PC12 Cells

**DOI:** 10.3390/ijms251910399

**Published:** 2024-09-27

**Authors:** Yu-Ning Jao, Yu-Jen Chao, Jui-Fen Chan, Yuan-Hao Howard Hsu

**Affiliations:** Department of Chemistry, Tunghai University, No. 1727, Sec. 4, Taiwan Boulevard, Xitun District, Taichung 40704, Taiwan; aa3522690@gmail.com (Y.-N.J.); zx85151@hotmail.com (Y.-J.C.); rivabox@hotmail.com (J.-F.C.)

**Keywords:** Parkinson’s disease, neurotransmitter, neurogenesis, neurodegeneration, dopaminergic metabolite, serotoninergic metabolite, glutamatergic metabolite, mass spectrometry

## Abstract

Parkinson’s disease (PD) affects movement; however, most patients with PD also develop nonmotor symptoms, such as hyposmia, sleep disorder, and depression. Dopamine levels in the brain have a critical influence on movement control, but other neurotransmitters are also involved in the progression of PD. This study analyzed the fluctuation of neurotransmitters in PC12 cells during neurogenesis and neurodegeneration by performing mass spectrometry. We found that the dopaminergic metabolism pathway of PC12 cells developed vigorously during the neuron differentiation process and that the neurotransmitters were metabolized into 3-methoxytyramine, which was released from the cells. The regulation of the intracellular and extracellular concentrations of adenosine indicated that adenine nucleotides were actively utilized in neural differentiation. Moreover, we exposed the differentiated PC12 cells to rotenone, which is a suitable material for modeling PD. The cells exposed to rotenone in the early stage of differentiation exhibited stimulated serotoninergic metabolism, and the contents of the serotoninergic neurotransmitters returned to their normal levels in the late stage of differentiation. Interestingly, the nondifferentiated cells can resist the toxicant rotenone and produce normal dopaminergic metabolites. However, when differentiated neuron cells were exposed to rotenone, they were seriously damaged, leading to a failure to produce dopaminergic neurotransmitters. In the low-dosage damage process, the amino acids that functioned as dopaminergic pathway precursors could not be absorbed by the cells, and dopamine and L-dopa were secreted and unable to be reuptaken to trigger the cell damage.

## 1. Introduction

Parkinson’s disease (PD) is a chronic neurodegenerative disease of the central nervous system, and its main symptoms are tremors, limb stiffness, and decreased motor function. Research indicates that the degeneration of the substantia nigra and dopamine-secreting neurons in the midbrain results in insufficient dopamine secretion in patients with PD [1]. Oxidative stress is one of the main causes of the degeneration of the substantia nigra and nerve cells [2]. Rotenone or paraquat inhibits intracellular mitochondrial respiratory chain complex I and causes an increase in oxidative stress, which leads to the selective degeneration of dopamine neurons in the substantia nigra and the accumulation of misfolded α-synuclein [3,4]. Rotenone is regarded as a suitable material for modeling PD in vivo and in vitro [5].

Current treatments for PD are aimed at replenishing dopamine levels in the brain. Levodopa (L-DOPA) is a precursor of dopamine, and its conversion into dopamine is catalyzed by aromatic amino acid decarboxylase. L-DOPA supplementation improves the symptoms of patients with PD [6]. Sinemet is a drug complex made of L-DOPA and carbidopa [7]. Because carbidopa is an inhibitor of dopamine decarboxylase, which degrades dopamine, the dosage of L-DOPA can be decreased to reduce the side effects of L-DOPA [8]. Unsaturated fatty acids have antioxidant and anti-inflammatory functions in protecting nerve cells and therefore have been used to treat PD. Dietary intake of omega-3 fatty acids by patients with PD helps to improve their depression symptoms [9].

In recent years, research on PD has focused on nonmovement disorders that occur in the early stage of PD or in mild PD [10]. Symptoms of PD appear before the onset of dopamine dyskinesia, and such symptoms include depression, apathy, anxiety, cognitive impairment, dementia, and attention deficit. Studies have indicated that cholinergic, serotonergic, glutamatergic, and noradrenergic pathways are involved in nonmotor disorders of PD; however, the exact neurotransmitter variation has not been studied [11]. Although dopamine deficiency is the target of current PD treatment research, dopamine is not the only neurotransmitter affected by PD. Serotonin, norepinephrine, and acetylcholine, which cause changes in mood, behavior, and cognition, are also affected by PD. The serotoninergic pathway is a critical neural signaling system Serotonin is associated with many types of psychopathological symptoms and is a drug target of antidepressants. Because the level of 5-hydroxyindoleacetic acid, which is the product of the oxidative deamination of serotonin, is very low in patients with PD, these patients are treated with serotoninergic agents [11].

Neurotransmitters are not only nerve signals but also regulators of the proliferation, differentiation, and survival of nerve cells [12]. In a previous study, the destruction of dopaminergic neurons in the substantia nigra of an adult mouse by using 6-hydroxydopamine reduced the neural precursor proliferation in the subventricular zone of the mouse by approximately 40%, which indicates that dopaminergic nigrostriatal projections regulate neural precursor proliferation [13]. Dopamine stimulates neostriatal neurogenesis and brain development by modulating the progenitor cell cycle of the lateral ganglionic eminence [14]. Serotonin is considered a developmental regulatory signal. The depletion of embryonic serotonin delays neurogenesis development, and serotonin promotes the differentiation of cortical and hippocampal neurons [15,16]. Moreover, 5-hydroxytryptamine hydrochloride (5-HT), norepinephrine, and acetylcholine are associated with the proliferation and differentiation of neural progenitor cells [17,18,19,20]. γ-Aminobutyric acid (GABA) is released mainly by interneurons but also by astrocytes [21]. In the subventricular zone of adult humans, the activation of GABA_A_ receptors mediates the inhibitory effect of GABA on the cell cycle, and the same mechanism is observed in the GABA-mediated control of embryonic stem cell proliferation [22,23].

Research on nerve transmission is currently restricted by limitations in analytical technology. Compared with other measurements, such as cyclic voltammetry and biosensing probes, mass spectrometers have higher sensitivity, selectivity, and signal-to-noise ratio and thus overcome the difficulty in detecting neurotransmitters in low concentrations and with low molecular weights [24,25,26,27]. In a previous study, automated solid-phase extraction liquid chromatography–mass spectrometry was used to analyze neurotransmitters and neuropeptides in blood [28]. Liquid chromatography–tandem mass spectrometry was successfully used to quantify the levels of the abnormal neurotransmitters 5-hydroxytryptamine, 5-hydroxyindoleacetic acid, and tryptophan in patients with Brunner syndrome [29]. In the present study, a comprehensive analysis of neurotransmitters and their metabolites was conducted through mass spectrometry to understand the interplay among metabolic pathways during PC12 differentiation and rotenone damage.

## 2. Results

### 2.1. Effects of Rotenone on the Morphology of Cells That Underwent Neurodifferentiation and Degeneration

The PC12 cells in the normal subculture were round and aggregated with nascent cells. We differentiated PC12 neuronal cells by using a low-serum culture with externally added NGF. Initially, the cell growth rate decreased, and after 3 days, the cells began to differentiate with the characteristics of neuronal cells and exhibited cell edge neurite connections and prominent nuclei. After 7 days of culture, the cells were characterized by intact neurons and exhibited robust neurite connection networks and enlarged bodies (Supplemental Figure S1). The pretreatment of undifferentiated PC12 cells with 100 nM rotenone inhibited their growth and differentiation. Cell growth was retarded for the first 4 days, and the cells stopped growing between the fourth and eighth days.

**Figure 1:**
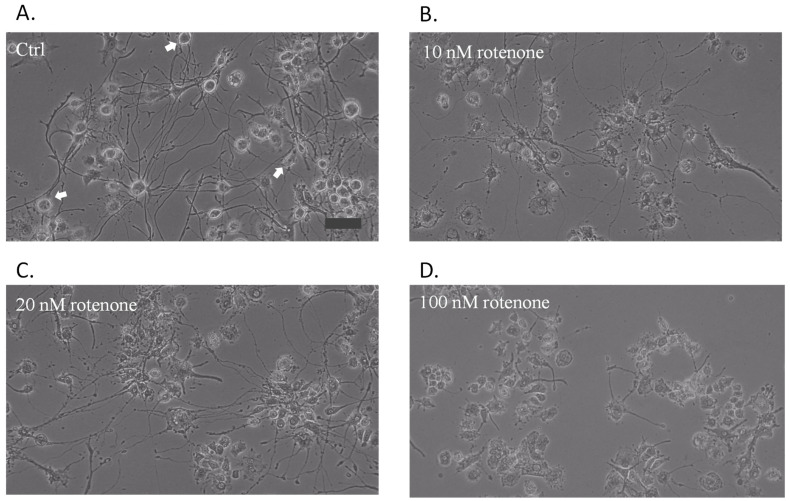
The effects of rotenone on the morphology PC12 differentiation: (**A**) PC12 cells were differentiated for 8 days. The arrow points out the neurite connections in the differentiated cell. The 6-day differentiated cells were treated with 10 nM (**B**), 20 nM (**C**), and 100 M (**D**) of rotenone for 48 h. The scale bar is 50 μm.

PC12 cells differentiated well in Opti-MEM containing 0.5% FBS and 50 nM NGF. On the eighth day of differentiation, the number of neurite connections had increased, neural networks were evident, and most of the cells were differentiated (Figure 1A). The differentiated cell showed connections with multiple neighboring cells. The differentiated neurons were suitable for neurodegeneration research and sensitive to rotenone. The treatment of these cells with 10 nM rotenone for 48 h resulted in the loss of the connection between neurites and morphological change (Figure 1B). After 48 h of treatment with 20 nM rotenone, obvious cell shrinkage was observed (Figure 1C). When the rotenone concentration was increased to 100 nM, the cells exhibited almost no neurite connection, and their nuclei were blurred; however, the cells could still become attached to the culture plate (Figure 1D). After treatment with rotenone, the axon length and neurite connections decrease as the concentration of rotenone increases. At the rotenone concentration of 20 nM, the connections between cells disappear. As the rotenone concentration reached 100 nM, the axon lost most of the axons.

### 2.2. Neurotransmitter Analysis

Q-TOF mass spectrometers have the advantage of accurate mass measurement, which is useful for finding biological metabolic molecules. A total of 51 metabolites with excellent signals and quality conforming to the Human Metabolome Database were selected from the identified mass spectra of the samples. All the selected mass spectra except tyramine, L-cysteine, and L-lysine had measurements with mass errors of less than 100 ppm (Supplemental Table S1). The neurotransmitters in the selected small metabolic molecules were subjected to further analysis. The strong mass spectrometry signals of ions were fragmented through tandem mass spectrometry. Moreover, the quantities and retention times of the small molecules were accurately determined. The purchased neurotransmitter standards and deuterated standards were used to acquire the tandem mass spectrum, which was compared with the spectra of the cellular samples and used to determine the appropriate collision energy.

The fragmentation pattern of each substance was used for molecular identification, and the fragmented ion with the highest intensity was usually selected for MRM semiquantification. Some catecholamines have the same phenol ions as product ions, and these catecholamines were preferentially selected for semiquantification (Supplemental Figure S2). A total of 17 neurotransmitters were identified on the basis of their mass-to-charge ratio, product ion pattern, and retention time (Table 1). Four deuterated standards were usually added to each sample, as listed in Table 1.

### 2.3. Activation of Dopaminergic Metabolism through PC12 Differentiation

**Figure 2:**
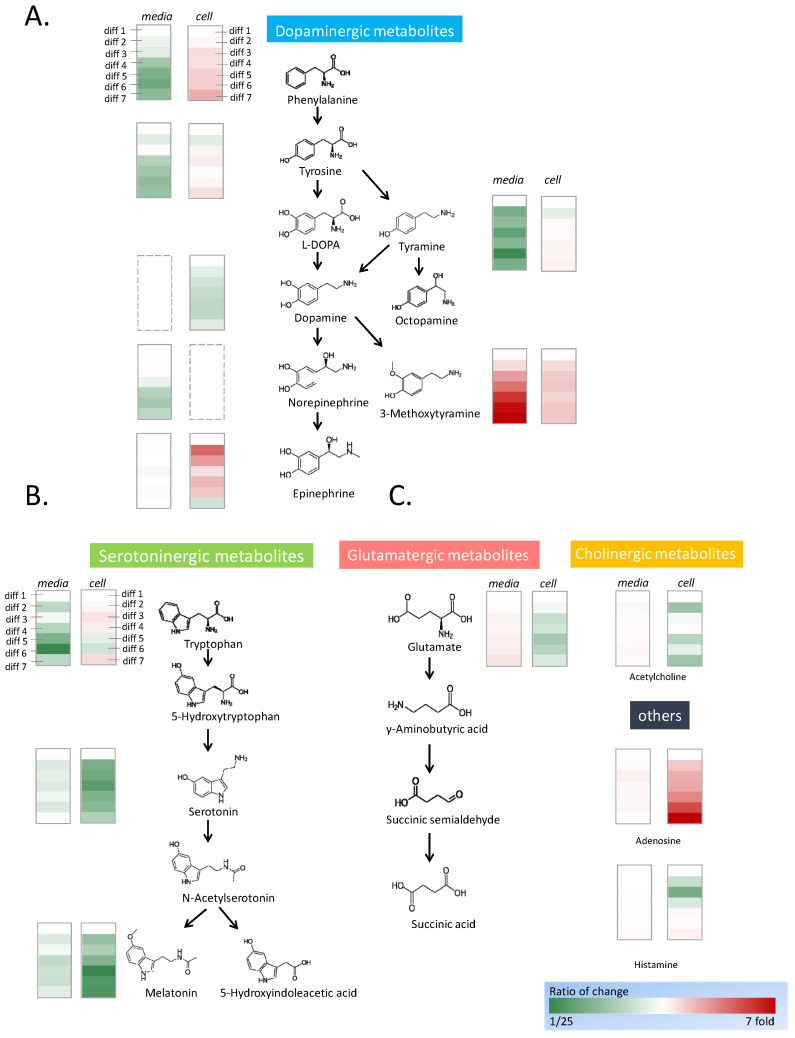
Fluctuation of neurotransmitters during neural differentiation. PC12 cells were stimulated by NGF for 7-day neural differentiation. Diff 1 to 7 indicate the number of differentiation days. The neurotransmitters were semiquantitated by mass spectrometry, and the fluctuation of the concentrations was calculated in folds. The concentrations of the neurotransmitters in the cells on the first day of differentiation are the control of the calculation. The concentrations of the neurotransmitters in the medium were the control to calculate the fold changes. The structure and the concentration changes in the dopaminergic metabolites (**A**), the serotoninergic metabolites (**B**), and other identified neurotransmitters (**C**) are shown in the figure. The increase in concentration is shown in red and the decrease in concentration is shown in green.

Thirteen neurotransmitters were detected in undifferentiated PC12 cells: phenylalanine, tyrosine, tyramine, dopamine, 3-methoxytyramine, epinephrine, tryptophan, serotonin, melatonin, glutamate, acetylcholine, adenosine, and histamine. With the progress of differentiation, the types and contents of neurotransmitters changed significantly. Because the culture medium contained some amino acids, their concentrations were measured as control values by using the mass spectrometer. During cell differentiation, 2 mL of the 5 mL culture medium was collected and replaced every day, and the concentrations of neurotransmitters in the medium were semiquantified and normalized against the day 1 control group. To examine the neurotransmitters inside the cells, seven dishes of the cells were differentiated and harvested at 1 to 7 days in triplicate. The neurotransmitters in the harvested cells were extracted and measured by mass spectrometer (Figure 2, Supplemental Table S2).

In the dopaminergic metabolic pathways, phenylalanine, tyrosine, and tyramine are upstream metabolized substances (Figure 2A). The concentrations of phenylalanine, tyrosine, and tyramine were high in the medium. With the progression of differentiation, the concentrations of phenylalanine and tyramine decreased significantly, whereas the concentration of tyrosine decreased marginally. The trends of the data were analyzed by linear regression to acquire the *p*-value in Microsoft Excel. The intracellular content of phenylalanine increased significantly, whereas those of tyrosine and tyramine increased marginally. The concentrations of the intermediate substances L-DOPA and dopamine were low, and these substances could not be detected in the cell culture medium throughout differentiation. The initial concentration of norepinephrine was high, and its concentration then decreased marginally after cell culture. Subsequently, the concentration of norepinephrine remained constant during cell differentiation. Inside the cell, the concentration of dopamine decreased significantly during the differentiation process. The end products, including epinephrine and 3-methoxytyramine showed significant accumulation during differentiation. Epinephrine did not exist in the original medium, and the epinephrine concentration was consistent during the differentiation process. The intracellular epinephrine concentration increased drastically in the early stage of differentiation and became flat with an increase in the differentiation time. The concentration of 3-methoxytyramine rose sharply in the medium with the progress of neuron differentiation; however, its intracellular concentration remained constant during neuron differentiation.

Serotoninergic metabolic pathways include tryptophan, a precursor of serotonin, serotonin, and melatonin, a metabolite of serotonin (Figure 2B). In the culture medium, the content of tryptophan was high and exhibited a downward trend after cell culture. During neuron differentiation, the tryptophan in the medium was absorbed by the cells; however, the concentration of intracellular tryptophan only marginally increased. The concentrations of serotonin and melatonin in the cells began to decrease significantly on the second day of differentiation. However, the concentrations of serotonin and melatonin in the medium were stable, which indicated they were not produced by the cells and secreted into the medium.

Adenosine is closely related to adenosine triphosphate (ATP) metabolism. The intracellular concentration of adenosine increased sharply with an increase in cell differentiation; however, its extracellular concentration was almost constant (Figure 2C). The intracellular concentrations of glutamate, acetylcholine, and histamine did not vary substantially with the amount of cell differentiation. With the progress of neuron differentiation, the extracellular concentrations of glutamate and acetylcholine increased marginally.

### 2.4. Effects of Rotenone Pretreatment on PC12 Differentiation

**Figure 3:**
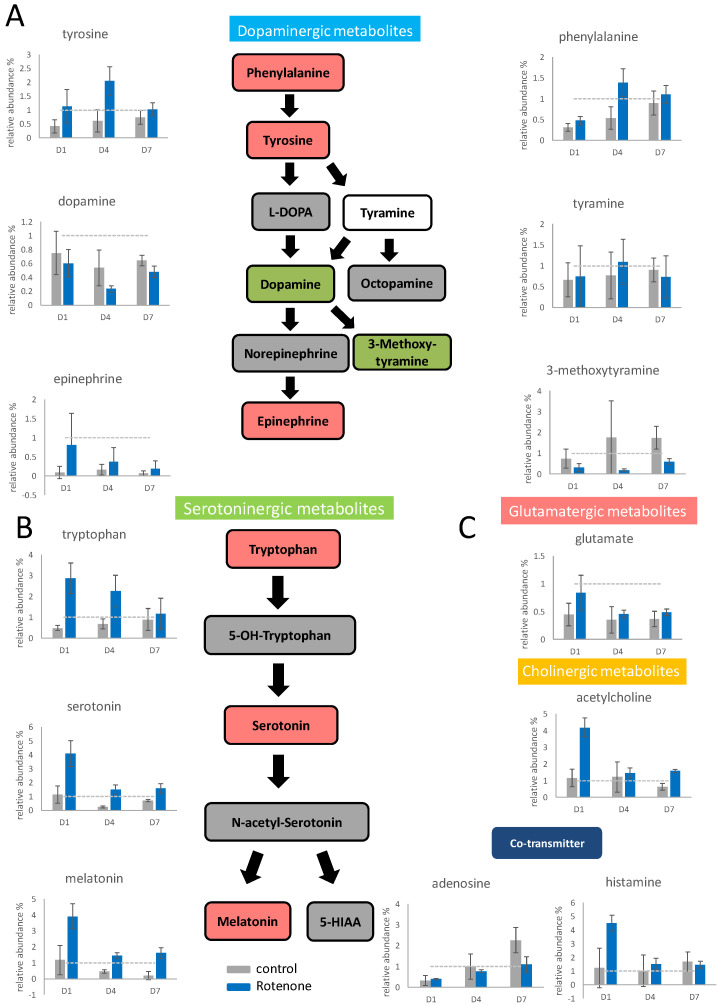
The neurotransmitter changes in PC12 upon rotenone pretreatment and NGF stimulation. PC12 was pretreated with 100 nM rotenone, and then the differentiation was stimulated by NGF for 7 days. The cells were harvested on the first, fourth, and seventh day. The neurotransmitters in the cells were extracted and semiquantitated by Q−TOF mass spectrometry. The results are shown in the bar graph. The gray bars indicate the control group without rotenone treatment, and the blue bars indicate the rotenone treated group. The experiments were triplicated, and the error bars are the standard deviation of the triplicates. In the metabolic pathways, green indicates a decreasing trend, and red indicates an increasing trend. The concentration changes in the dopaminergic metabolites (**A**), the serotoninergic metabolites (**B**), and other identified neurotransmitters (**C**) are shown in the figure. The dash line in the bar graph shows the concentration level in the control group.

To evaluate the effects of rotenone on neuron differentiation, PC12 cells were pretreated with 100 nM rotenone for 24 h and then supplemented with NGF to initiate differentiation. The concentrations of the intracellular neurotransmitters were determined through mass spectrometry (Supplemental Table S3), and significant variations in dopaminergic metabolite concentrations were found (Figure 3A). Slight elevation was discovered for the upstream substances phenylalanine and tyrosine, especially on the fourth day of differentiation. The intermediate metabolite dopamine slightly decreased under the influence of rotenone, especially on the fourth day of differentiation. After rotenone treatment, the content of epinephrine increased significantly; however, the content of the other end product affected by rotenone, namely 3-methoxytyramine, significantly decreased the most on the fourth day in the 7-day differentiation. The major end product in the dopaminergic pathway shifted from 3-methoxytyramine to epinephrine upon pretreatment with rotenone.

The concentrations of tryptophan, serotonin, and melatonin (serotoninergic metabolites) increased significantly after rotenone pretreatment, and their contents increased sharply, especially on the first day of differentiation (Figure 3B). Glutamate accumulated under the influence of rotenone on the first day of differentiation, and its content then returned to normal (Figure 3C). The acetylcholine content on the first day of differentiation under the influence of rotenone was four times higher than that in the control group; however, the acetylcholine content returned to normal after the fourth day of differentiation. The adenosine content increased slower after rotenone treatment. The histamine content increased fourfold on the first day of treatment but then returned to normal after the fourth day of treatment.

### 2.5. Effects of Rotenone Pretreatment on Neurotransmitter Release

Rotenone can damage nerve cells and may cause these cells to release altered amounts of neurotransmitters. To evaluate the neurotransmitter release effects of rotenone, rotenone-pretreated PC12 cells were differentiated through NGF induction, culture medium samples were collected every 24 h, and the neurotransmitter contents were determined through mass spectrometry (Supplemental Figure S3). Overall, rotenone pretreatment had little effect on the release of neurotransmitters. The extracellular concentrations of the dopaminergic upstream metabolites phenylalanine and tyrosine, as well as the downstream metabolite 3-methoxytyramine, increased. The extracellular concentration of the serotoninergic upstream metabolite tryptophan increased to a greater extent than in the regular differentiated cells, whereas the serotonin and melatonin contents did not change significantly. Under the influence of rotenone, the extracellular accumulation of adenosine was high, whereas the extracellular histamine content decreased.

### 2.6. Effects of Rotenone on Neurotransmitters during Neurodegeneration

**Figure 4:**
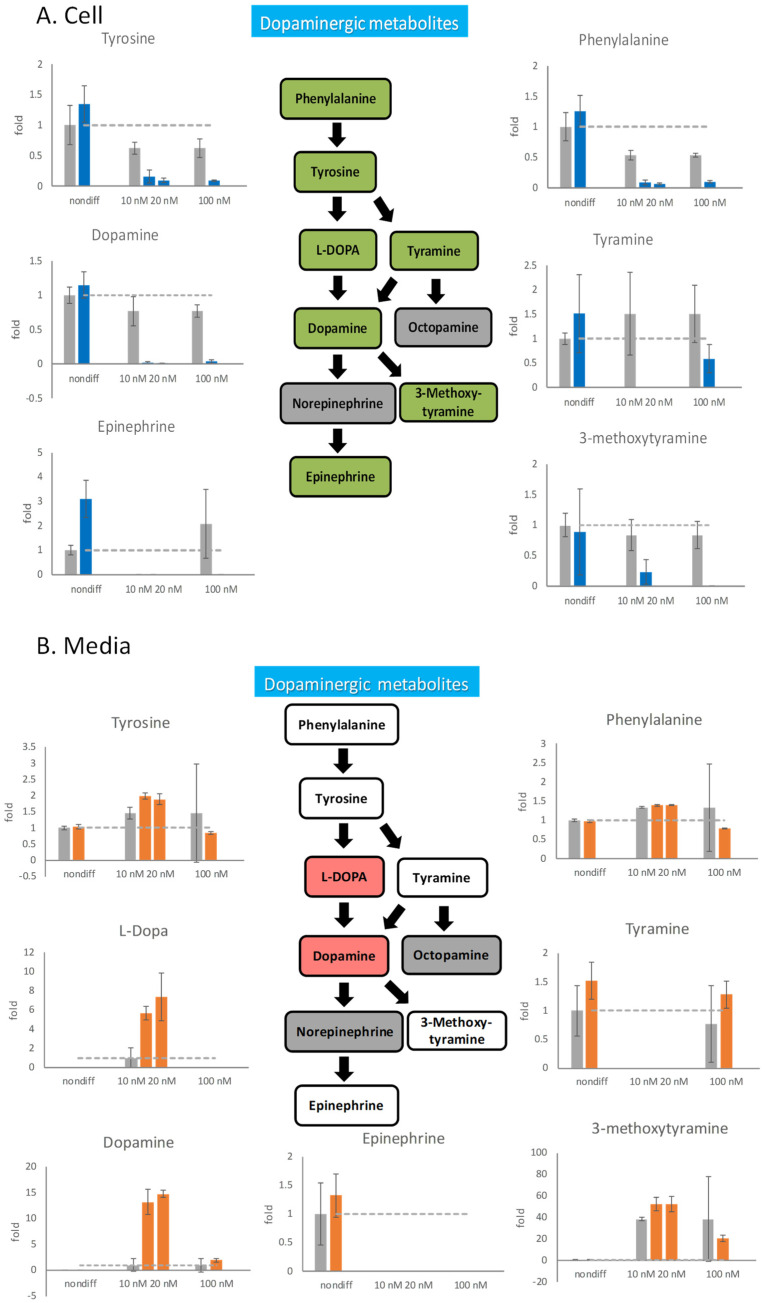
The effects of rotenone on dopaminergic neurotransmitters during neurodegeneration. PC12 cells were differentiated for 6 days. After the cells were treated with 10 nM, 20 nM, and 100 nM rotenone for 48 h, the cells and the media were harvested. The neurotransmitters in the cell (**A**) and in the media (**B**) were semiquantitated by mass spectrometry. In the metabolic pathways, green indicates a decreasing trend, and red indicates an increasing trend. The gray bars are the control group, and the blue and orange bars are rotenone-treated groups. The experiments were triplicated. The intracellular concentration level in the rotenone treated group is shown in blue, the extracellular concentration level in the rotenone treated group is shown in orange, and the control group is shown in gray. The dash line in the bar graph shows the concentration level in the control group.

Rotenone not only perturbs neuron differentiation but also damages differentiated cells. Six days after differentiation, 100 nM rotenone was added to this medium for 48 h to initiate the cellular damage. The neurotransmitters in the cells and medium were measured by mass spectrometry (Supplemental Table S4). Observations by mass spectrometry indicated that all intracellular neurotransmitters discontinued their metabolization because of the influence of rotenone (Figure 4A). Because 100 nM rotenone excessively damages the cells and most neurotransmitter concentrations drop to 0, the progression of nerve atrophy cannot be observed in this experimental condition. Therefore, 10 and 20 nM rotenone was tested in our experiment. Even these low concentrations of rotenone led to the synaptic degeneration of nerve cells. The intracellular concentrations of all dopaminergic neurotransmitters decreased to 0, which indicated that rotenone inhibited the metabolism of these neurotransmitters.

Because rotenone inhibited the metabolism of intracellular neurotransmitters, we speculated that neurotransmitters released outside cells should also be affected by rotenone. By measuring changes in the concentrations of extracellular neurotransmitters, how nerve cells respond through neurotransmitters when they undergo degeneration can be determined. We investigated the effects of 100 nM rotenone on the release of neurotransmitters from undifferentiated PC12 cells and 6-day-differentiated neural cells for 48 h (Figure 4). Undifferentiated cells were treated with 100 nM rotenone, and the release of neurotransmitters was not considerably different after rotenone treatment. However, after 6 days of differentiation, 100 nM rotenone was added for 48 h, and the release of neurotransmitters was drastically affected. The extracellular content of phenylalanine and tyrosine in the rotenone-treated cells was 30% lower than that in the control cells, and the extracellular content of tyramine in the rotenone-treated cells was 15% higher than that in the control cells. The release of dopamine was significantly affected by 100 nM rotenone, and the dopamine content in the rotenone-treated cells was approximately twice that in the control cells. However, the release of the downstream product 3-methoxytyramine in the experimental group decreased. Although changes in metabolite contents could be observed, the standard errors were relatively large.

After decreasing the rotenone concentration to 10 and 20 nM, the extracellular content of phenylalanine, which is an upstream dopaminergic metabolite, remained unchanged, whereas that of tyrosine increased (Figure 4B). The midstream substances L-DOPA and dopamine were drastically affected by the low concentration of rotenone and exhibited six to eight folds of increased extracellular concentrations. The extracellular accumulation of the final product 3-methoxytyramine also increased under rotenone concentrations of 10 and 20 nM.

**Figure 5:**
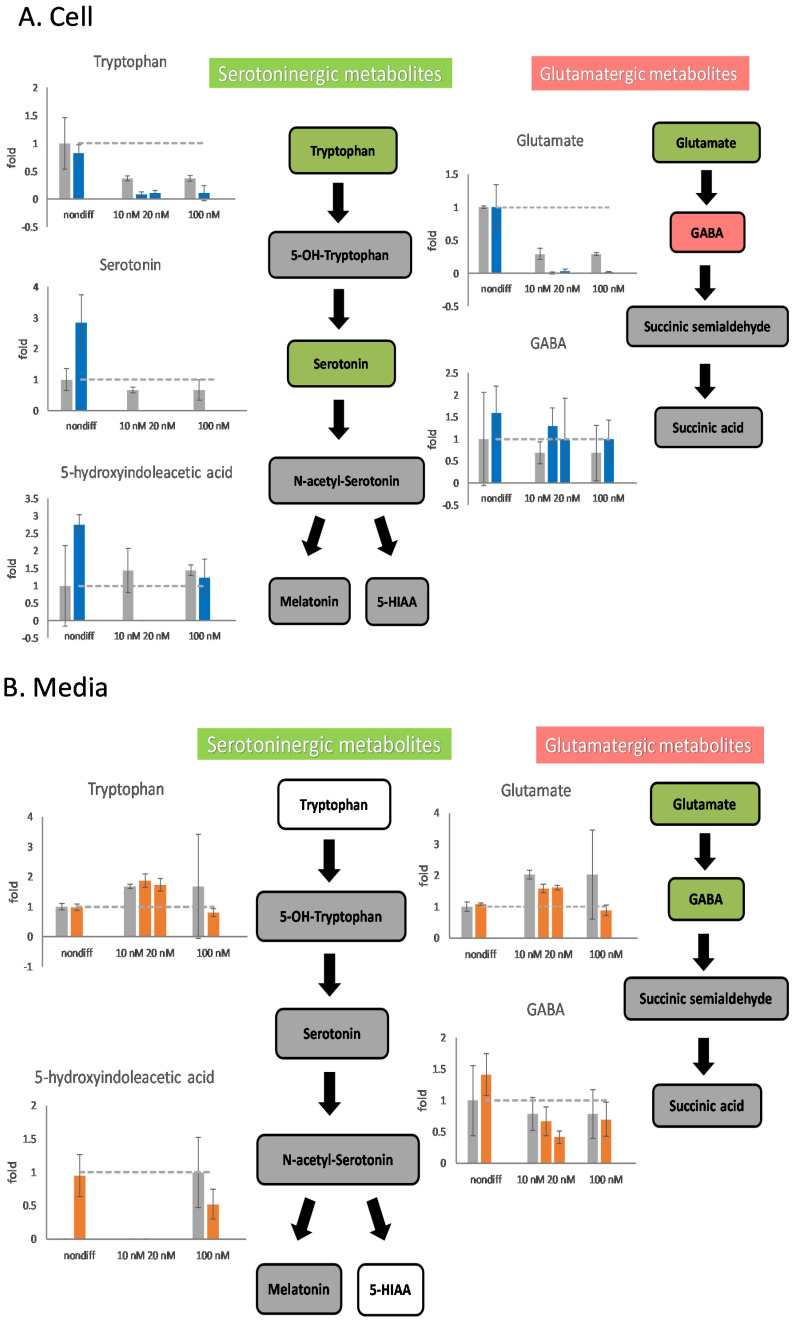
The effects of rotenone on serotoninergic and glutamatergic neurotransmitters during neurodegeneration. PC12 cells were differentiated for 6 days. After supplementing 10 nM, 20 nM, and 100 nM rotenone for 48 h, the cells and the media were harvested. The neurotransmitters in the cell (**A**) and in the media (**B**) were semiquantitated by mass spectrometry. In the metabolic pathways, green indicates a decreasing trend, and red indicates an increasing trend. The experiments were triplicated. The intracellular concentration level in the rotenone treated group is shown in blue, the extracellular concentration level in the rotenone treated group is shown in orange, and the control group is shown in gray. The dash line in the bar graph shows the concentration level in the control group.

Undifferentiated cells affected by 100 nM rotenone exhibited little damage or stimulated the metabolism of serotoninergic neurotransmitters (Figure 5). The intracellular contents of serotonin and 5-HIAA increased by a factor of more than 2.5 after the addition of 100 nM of rotenone. When rotenone concentrations of 10 and 20 nM were used as a treatment for neurodegeneration, the serotoninergic metabolic pathway was affected and stopped intracellular metabolism after differentiation (Figure 5A). The differentiated cells in the serotoninergic pathway were affected by 10–100 nM rotenone, which caused a decrease in the extracellular content of 5-HIAA.

When 10 or 20 nM rotenone was used for cell treatment, the metabolism of glutamatergic metabolite was affected. The intracellular glutamate content was almost 0; however, the GABA content increased (Figure 5A). In the glutamatergic pathway, the intracellular and extracellular concentrations of glutamate decreased when using 10, 20, or 100 nM rotenone for treatment (Figure 5). The extracellular GABA content in the samples treated with 20 nM rotenone was lower than that in the control samples (Figure 5B). It is important to note that the concentration of GABA in the mass spectrometer was low, and the standard deviation of the GABA measurement was large.

**Figure 6:**
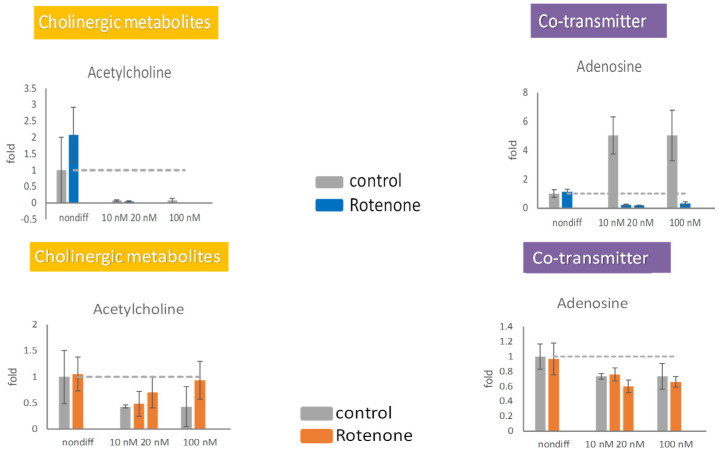
The effects of rotenone on acetylcholine and adenosine during neurodegeneration. PC12 cells were differentiated for 6 days. After supplementing 10 nM, 20 nM, and 100 nM rotenone for 48 h, the cells (**upper** panels) and the media (**lower** panels) were harvested. The neurotransmitters in the cell and in the media were semiquantitated by mass spectrometry. The experiments were triplicated. The intracellular concentration level in the rotenone treated group is shown in blue, the extracellular concentration level in the rotenone treated group is shown in orange, and the control group is shown in gray. The dash line in the bar graph shows the concentration level in the control group.

The extracellular concentration of acetylcholine increased in the presence of 20 and 100 nM rotenone (Figure 6). The extracellular concentration of adenosine was not significantly affected by rotenone. The intracellular production of adenosine in the differentiated cells was completely stopped by rotenone, whereas the extracellular concentration of adenosine remained stable (Figure 6). Differentiated cells were unable to retain acetylcholine inside the cell; however, the release of acetylcholine increased under treatment with 20 or 100 nM rotenone.

## 3. Discussion

### 3.1. Activation of Dopaminergic Metabolites during Neuron Differentiation

Neurite growth considerably changes cell morphology and causes the expression of neuronal protein biomarkers; thus, neurite growth can indicate the differentiation of PC12 cells [30]. The types and contents of neurotransmitters during neuron differentiation in the PC12 cellular model have been used to study neurodifferentiation and neurotoxicity [31]. Our results coordinate well with previous studies in which neuron differentiation had a significant effect on neurosecretion and neurotransmitter content [32]. Some monoamine neurotransmitters are metabolites of the same amino acids; therefore, these neurotransmitters were grouped into dopaminergic and serotoninergic metabolites [33]. PC12 differentiation stimulates the synthesis of dopaminergic metabolites and inhibits the metabolism from shifting toward serotoninergic metabolites.

The intracellular contents of 3-methoxytyramine and epinephrine increased during neuron differentiation but at different times. The epinephrine content changed drastically in the early stage of neuron differentiation and then remained constant with a further increase in the differentiation time. The end product 3-methoxytyramine was secreted out of the cells during neuron differentiation, and its content increased sharply in the culture medium in the later stages of differentiation [34]. Neuron differentiation activated dopaminergic metabolites but suppressed serotoninergic metabolites. The activation of dopaminergic metabolites was accompanied by a sharp increase in the intracellular adenosine concentration, which further regulates the adenosine receptors [35,36]. Overall, this study demonstrated that neuron differentiation significantly activates dopaminergic neurotransmitters.

### 3.2. Rotenone Treatment Shifts Dopamine Metabolism and Activates Serotoninergic Pathway

Rotenone pretreatment significantly affected the contents of dopaminergic metabolites in PC12 cells. Specifically, this pretreatment caused a shift from 3-methoxytyramine activation to epinephrine activation in dopamine metabolism, which could be a death signal to the differentiated cell [37]. Cell death was not evident with 10, 20, and 100 nM rotenone treatments, but detached cells were slightly observable with 100 nM rotenone. Under rotenone pretreatment, the extracellular content of 3-methoxytyramine remained constant; however, its intracellular content increased. Moreover, the intracellular epinephrine content increased upon pretreatment of rotenone. The aforementioned results suggest that rotenone treatment significantly accelerates the conversion of dopamine to downstream metabolites during neuron differentiation.

Furthermore, this study indicated that rotenone pretreatment caused increases in the contents of serotoninergic metabolites such as tryptophan, serotonin, and melatonin, particularly on the first day of neuron differentiation. Rotenone caused a toxicant shock to the cells, and many neurotransmitters were elevated immediately, including epinephrine, serotoninergic metabolites, glutamate, acetylcholine, and histamine, and then returned to their normal level. The results suggest that rotenone treatment affects the production of other neurotransmitters apart from dopamine during the differentiation of PC12 cells. The production of the serotoninergic neurotransmitters may have a protective effect against rotenone toxicity [38]. Interestingly, there was a decrease in the adenosine content upon rotenone treatment, which might have resulted from the inhibition of electron transport complex I by rotenone.

### 3.3. Detrimental Effect of Rotenone on Differentiated PC12 Cells

Research has indicated that differentiated PC12 cells are susceptible to rotenone damage [39], as well as the dopaminergic neurons in rats [40]. Our investigation showed that 100 nM of rotenone seriously damaged the metabolism of dopaminergic neurotransmitters in differentiated cells but only slightly affected the nondifferentiated cells. Therefore, rotenone treatment resulted in more significant decreases in the neurotransmitter concentrations of the differentiated PC12 cells. In rats, rotenone damage is not limited to dopamine; the effects were predominantly both associated with serotonin and dopamine [41]. All the neurotransmitter contents except for the extracellular dopamine and L-DOPA contents did not exhibit considerable changes when the rotenone concentration was decreased from 100 to 10 nM. The intracellular dopamine and L-DOPA contents remained low, but the extracellular dopamine and L-DOPA contents drastically elevated, which indicated that the production and secretion of dopamine and L-DOPA were stimulated upon rotenone treatment. The endogenous dopamine has been shown to aggravate rotenone-induced toxicity [42]. Secreted dopamine and L-DOPA could neither be further metabolized into epinephrine nor recycled back to the cells. Glutamate serves as an anaplerotic substrate to replenish tricarboxylic acid (TCA) cycle intermediates. Rotenone treatment causes the depletion of glutamate, which will have an inhibitory effect on the TCA cycle.

In conclusion, the results of this study indicate the significant effects of neuron differentiation and rotenone treatment on the metabolism of neurotransmitters in PC12 cells. Neuron differentiation activated the metabolism of dopaminergic neurotransmitters, which led to increases in the intracellular contents of 3-methoxytyramine and epinephrine but the suppression of the production of serotoninergic metabolites. By contrast, rotenone treatment stimulated the production of dopamine metabolites, particularly epinephrine, and also affected the production of other neurotransmitters, such as serotonin, melatonin, glutamate, acetylcholine, and histamine. Furthermore, rotenone was detrimental to differentiated cells and led to declines in neurotransmitter concentrations and the inability to recycle or further metabolize dopamine and L-DOPA. The findings of this study shed light on the intricate regulation of neurotransmitter metabolism during cell differentiation and the potentially harmful effects of rotenone exposure for PC12 cells.

## 4. Materials and Methods

### 4.1. Materials

Acetylcholine, (*S*)-2-amino-3-hydroxypropionic acid (L-serine), dopamine hydrochloride, dopamine-d_4_, 3,4-dihydroxy-α-(methylaminomethyl)benzyl alcohol (epinephrine), GABA, glutamic acid-d_5_, histamine, 5-hydroxyindole-3-acetic acid (HIAA), 5-HT (serotonin), poly-L-lysine, L-tyrosine, melatonin, norepinephrine hydrochloride, norepinephrine-d_6_, and nerve growth factor (NGF) were purchased from Sigma-Aldrich (St. Louis, MO, USA). Furthermore, adenosine-2-d_1_ was purchased from CDN Isotopes (Vaudreuil, QC, Canada), and L-DOPA was purchased from U.S. Pharmacopeia. PC12 (rat adrenal medulla pheochromocytoma) cells were purchased from ATCC (CRL-1721; Rockville, MD, USA), and rotenone was purchased from Merck Millipore (Guyancourt, France). Finally, Opti-MEM medium, RPMI 1640 medium, fetal bovine serum (FBS), and horse serum (HS) were purchased from Gibco (Grand Island, NY, USA). The Ultra-high-performance liquid chromatography (UHPLC) is from Thermo Fisher Scientific Ultimate 3000, Dionex, Idstein, Germany. The micrOTOF-Q III (ESI-Q-TOF) is from Bruker Company, Ettlingen, Germany.

### 4.2. PC12 Cell Culture and Differentiation

PC12 cells were cultured on a poly-L-lysine-coated plate to facilitate the adhesion of the cells. A total of 800 μL of 0.1 mg/mL polylysine solution was spread evenly on a culture plate of size 6 cm. The plate was air-dried for 10 min and then washed with 1 mL of sterile water to remove excess polylysine. All the culture plates containing the aforementioned solution were sterilized through ultraviolet exposure, sealed with parafilm, and stored at 4 °C.

The cell culture medium was composed of 85% RPMI 1640 medium, 10% heated HS, 5% FBS, and 1% penicillin/streptomycin solution. The cells were cultured in a 5% CO_2_ incubator at 37 °C, and the cell confluence on the plate was controlled between 30% and 70%. To differentiate PC12 cells, 10^6^ cells were placed in a 6 cm culture dish in 4 mL of low-serum cell culture medium containing 4 μL of 100 ng/mL NGF. The low-serum cell culture medium contained 98% RPMI 1640 medium, 1% heated HS, 1% FBS, and 1% PS. The medium with 100-ng/mL NGF was replaced every 48 h, and the cells differentiated within approximately 4 days.

To examine the release of the neurotransmitters to the medium during PC12 differentiation, three 10 cm culture dishes of PC12 cells were differentiated, 2 mL of the 5 mL culture medium was collected and replaced every day, and the concentrations of neurotransmitters in the medium were semiquantified and normalized against day 1 control group. To examine the neurotransmitters inside the cells, 7 dishes of the cells were differentiated and harvested at 1 to 7 days in triplicate. The neurotransmitters in the harvested cells were extracted as 4.5.

### 4.3. Inhibition of Cell Differentiation by Rotenone

A total of 6.3 × 10^5^ cells were placed in a 10 cm culture dish and cultured in 5 mL of low-serum medium containing 100 nM NGF. The experimental group was supplemented with 100 nM rotenone dissolved in dimethyl sulfoxide (DMSO), and the control group was supplemented with the same amount of DMSO. A total of 2 mL of medium was collected every 24 h and replaced with fresh medium containing 100 nM NGF. On the first, fourth, and seventh days of the differentiation period, the cellular morphology was recorded, and the culture medium and cells were collected for subsequent neurotransmitter analysis.

### 4.4. Effects of Rotenone on Neurodegeneration

To optimize the neural differentiation conditions, the low-serum cell culture medium used in the experiment contained 98.5% Opti-MEM, 0.5% FBS, and 1% PS. Before cells were differentiated, 6.3 × 10^5^ cells were placed in a 10 cm culture dish with 5 mL of culture medium and 50 nM NGF. The medium containing 50 nM NGF was renewed every 48 h. On the sixth day of cell differentiation, 10, 20, or 100 nM rotenone with DMSO was added to the experimental group, and 0.1 μL of DMSO was compensated to the control group. After 48 h, the cellular morphology was recorded using a microscope, and the culture medium and cells were collected for subsequent neurotransmitter analysis.

### 4.5. Extraction of Neurotransmitters in Cells and Medium

To extract neurotransmitters from cells, 300 μL of 0.3% formic acid and 400 ng of deuterated neurotransmitter standards were added to the samples. Cells were disrupted through sonication at 60% amp (Q125 Sonicator, Qsonica, Newtown, CT, USA; 125 W) for 10 s on ice. The samples were centrifuged at 15,000 rpm for 10 min at 4 °C, following which, 250 μL of supernatant was collected and centrifuged at 15,000 rpm at 4 °C. Subsequently, 200 μL of supernatant was filtered using a 10,000-KDa centrifugal molecular weight cutoff (MWCO) filter (PALL, New York, NY, USA) at 14,000× *g* for 5 min at 4 °C. The prepared samples were then examined under a mass spectrometer.

To acidify 200 μL of the cell culture medium, 0.3% formic acid was added to this medium. The medium was then centrifuged at 15,000 rpm for 10 min at 4 °C. Subsequently, the supernatant was diluted 10-fold using 0.3% formic acid. A total of 400 ng of deuterated neurotransmitter standards was added to the samples. The supernatant was filtered using the PALL MWCO filter at 14,000× *g* for 5 min at 4 °C. The samples were then subjected to mass spectrometry analysis.

### 4.6. Liquid Chromatography–Mass Spectrometry Analysis

Ultra-high-performance liquid chromatography (UHPLC, Thermo Fisher Scientific Ultimate 3000, Dionex, Idstein, Germany) and ESI-Q-TOF (micrOTOF-Q III, Bruker Company, Germany) were used to analyze neurotransmitters. For each analysis, 20 μL of sample was injected into UHPLC. Reverse-phase chromatography was conducted at 30 °C by using an Acclaim RSLC120 C18 column. Mobile phase A was 0.3% formic acid, and mobile phase B was acetonitrile containing 0.3% formic acid. The flow rate of the mobile phase gradient was 0.075 mL/min. The percentage of mobile phase A was maintained at 100% for 4 min, then decreased from 100% to 90% over 5 min, then decreased again from 90% to 0% over 6 min, and finally kept at 0% for 7 min. A Quadrupole Time-of-Flight (Q-TOF) mass spectrometer was used in the positive mode to detect ions, and the mass range was set as 80–300 *m*/*z*. Mass spectrometer fragmentation and product ion detection were performed in three time sections under multireaction monitoring (MRM). MRM parameters for fragmentation differ according to the properties of the substances.

### 4.7. Quantification and Semiquantification of Neurotransmitters

Four neurotransmitters, namely glutamate, dopamine, norepinephrine, and adenosine, had deuterated internal standards. Therefore, the absolute quantitative concentrations were calculated by comparing the areas of the peaks of neurotransmitters to those of the deuterated standards. The number of cells was recorded when the cells were harvested and used to calculate the number of neurotransmitters per cell. For the remaining neurotransmitters without deuterated standards, only folds relative to the control group are presented. In Figure 3, Figure 4 and Figure 5 and Supplemental Figure S3, a *t*-test was conducted to assess significance. If significant differences were found, the pathway boxes were colored red for increasing concentrations and green for decreasing concentrations. If the changes did not meet the *t*-test criteria but all three treatment averages were consistently higher or lower than the control, the boxes were still colored accordingly.

### 4.8. Statistical Analysis

The experiments in this study were conducted in triplicate, and the experimental results were analyzed using the one-tailed Student’s *t*-test. Significance was examined at levels of <0.05, <0.01, and <0.001.

## Figures and Tables

**Table 1:** Identification of neurotransmitters in PC12 cells by MS/MS.

No.	Observed Mass *m/z*	Identified Mass *m/z*	Neurotransmitter	Retention Time (min)	Section (min)	Collision (eV)
1	104.07	87	γ-Aminobutyric acid	4.0–4.8	I (0–14)	30
2	112.08	95	Histamine	3.7–4.8	I (0–14)	30
3	146.11	87	Acetylcholine	5.9–9.0	I (0–14)	20
4	148.06	84	Glutamate	4.7–5.9	I (0–14)	25
5	153.09	88	Glutamate-D5	4.7–5.9	I (0–14)	25
6	154.08	91	Dopamine	9.4–12.0	I (0–14)	25
7	158.11	95	Dopamine-D4	9.4–12.0	I (0–14)	25
8	170.08	152	Norepinephrine	4.8–5.6	I (0–14)	25
9	176.11	158	Norepinephrine-D6	4.8–5.6	I (0–14)	25
10	184.09	166	Epinephrine	5.9–8.0	I (0–14)	25
11	138.05	121	Tyramine	19.7–20.1	II (15–25)	30
12	166.08	120	Phenylalanine	19.4–20.3	II (15–25)	25
13	168.10	91	3-Methoxytyramine	17.7–18.7	II (15–25)	30
14	177.09	160	Serotonin	18.1–19.0	II (15–25)	25
15	182.08	91	Tyrosine	16.1–17.0	II (15–25)	30
16	192.05	146	5-Hydroxyindoleacetic acid	21.3–21.9	II (15–25)	30
17	198.13	152	L-dopa	12.6–15.0	II (15–25)	30
18	205.08	188	Tryptophan	20.6–21.0	II (15–25)	30
19	233.12	174	Melatonin	22.1–23.5	II (15–25)	30
20	268.09	136	Adenosine	16.5–17.3	II (15–25)	20
21	269.10	137	Adenosine-D1	16.5–17.3	II (15–25)	20

## Data Availability

The original contributions presented in the study are included in the article/supplementary material, further inquiries can be directed to the corresponding author.

## Supplementary Materials

The following supporting information can be downloaded at https://www.mdpi.com/article/10.3390/ijms251910399/s1.
