# Supplementary material for: Mass Spectrometry Analysis of Neurotransmitter Shifting during Neurogenesis and Neurodegeneration of PC12 Cells
Source: Int J Mol Sci. 2024 Sep 27;25(19):10399. doi: 10.3390/ijms251910399 (PMC11477332; doi:10.3390/ijms251910399)
Supplement: Supplementary file 1 [file ijms-25-10399-s001.zip › ijms-3126972-supplementary.pdf]

Supplemental Table S1. Identification of metabolites in PC12 cells.

No.	Metabolites	Observed mass (m/z)	Theoretical mass (m/z)	Error (ppm)	MRM RT(min)
1	glycine	76.0448	76.03985	65.1	
2	alanine	90.053	90.0555	-27.8	
3	GABA	104.0737	104.07115	24.5	3.2-4.8
4	choline	104.1051	104.10754	-23.4	
5	serine	106.0496	106.05042	-7.7	
6	histamine	112.0877	112.0796473	71.8	3.7-4.8
7	L-pyroline-5-carboxylic acid	114.0544	114.0555	-9.6	
8	proline	116.0708	116.07115	-3.0	
9	L-valine	118.0857	118.0868	-9.3	
10	threonine	120.0661	120.06607	0.2	none
11	L-cysteine	120.986	121.0197492	-278.9	
12	5-oxo-proline	130.05	130.05042	-3.2	12.2-12.9
13	L-isoleucine	131.0886	131.0946287	-46.0	
14	leucine	132.1016	132.10245	-6.4	15-17.1
15	aspartic acid	134.0446	134.04533	-5.4	4.7-5.3
16	tyramine	138.0547	138.084064	-212.7	19.7-20.1
17	2-propyl-2,4-pentadienoic acid	141.0848	141.09156	-47.9	10-10.9
18	L-lysine	146.1368	146.1055277	214.0	
19	acetylcholine	146.1182	146.1181	0.7	5.9-9.0
20	glutamine	147.0758	147.07697	-8.0	
21	glutamate	148.0601	148.06098	-5.9	4.7-5.9
22	D5-glutamate	153.0905	153.104305	-90.2	4.7-5.9
23	dopamine	154.0854	154.0868	-9.1	9.4-12.0
24	L-histidine	156.0733	156.0773	-25.6	
25	D4-dopamine	158.1106	158.12146	-68.7	9.4-12.1
26	succinylacetone	159.0619	159.06573	-24.1	none
27	2-amino-4-methylenepentanedioic acid	160.0761	160.06098	94.5	none
28	3-methyldioxyindole	164.068	164.07115	-19.2	
29	phenylalanine	166.0864	166.0868	-2.4	19.4-20.3
30	3-methoxytyramine	168.1002	168.10245	-13.4	17.7-19.0
31	norepinephrine	170.0794	170.08172	-13.6	4.8-5.6
32	L-arginine	174.11	174.1116757	-9.6	

**33**	suberic acid	175.0929	175.09704	-23.6	5.2-5.9
**34**	D6-norepinephrine	176.1172	176.13371	-93.7	4.8-5.6
**35**	n-carboxyethyl-g-aminobutyric acid	176.0899	176.09228	-13.5	
**36**	serotonin	177.101	177.094963	34.1	18.1-19.0
**37**	adrenochrome	180.0709	180.06607	26.8	21.3-21.8
**38**	tyrosine	182.0793	182.08172	-13.3	16.1-17.0
**39**	epinephrine	184.0955	184.09737	-10.2	5.9-8.0
**40**	L-dopa	198.0767	198.0688078	39.8	12.6-15.0
**41**	L-acetylcarnitine	204.1196	204.12358	-19.5	
**42**	tryptophan	205.0823	205.0898776	-36.9	20.5-21.2
**43**	n-acetylgalactosamine	222.0943	222.09776	-15.6	
**44**	4,5-dihydrovomifoliol	227.1605	227.16472	-18.6	none
**45**	suberylglycine	232.1176	232.1185	-3.9	
**46**	butyrylcarnitine	232.1681	232.15448	58.7	none
**47**	melatonine	233.1259	233.1211778	20.3	22.1-23.5
**48**	hexanoylcarnitine	260.1906	260.18618	17.0	none
**49**	adenosine	268.0993	268.0967539	9.5	16.5-17.3
**50**	D1-adenosine	269.1057	269.1054189	1.0	16.5-17.3
**51**	hydroxyhexanoycarnitine	276.1905	276.1811	34.0	none

Supplemental Table S2. Intracellular and extracellular neurotransmitter differences during the 7-day differentiation (Diff.1-7) process. The concentrations of neurotransmitters were normalized to the neurotransmitter concentrations on day 1. The p-values are the results from statistical analysis against Diff.1 results.

Compounds	*Ratio±SD*	*Diff.1*	*Diff.2*	*Diff.3*	*Diff.4*	*Diff.5*	*Diff.6*	*Diff.7*
Phenylalanine	***Media***	1.00±0.03	0.93±0.11	0.91±0.13	0.74±0.07	0.65±0.13	0.62±0.09	0.70±0.15
	P value#				0.0001***			
	Cell	1.00±0.29	1.22±0.86	1.82±0.99	1.72±0.87	2.10±1.62	2.10±0.19	2.88±0.93
	P value	_	0.9370	0.3467	0.3296	0.2601	0.0131*	0.0157*
Tyrosine	***Media***	1.00±0.24	0.90±0.26	0.97±0.38	0.79±0.28	0.74±0.27	0.71±0.17	0.73±0.22
	P value#				0.1230			
	Cell	1.00±0.59	0.85±0.60	1.25±0.92	1.46±0.96	1.05±0.92	1.23±0.17	1.77±0.60
	P value	_	0.7114	0.9929	0.7063	0.8788	0.4276	0.1788
Dopamine	***Media***	ND	ND	ND	ND	ND	ND	ND
	P value#				_			
	Cell	1.00±0.42	0.87±0.40	0.78±0.38	0.72±0.34	0.69±0.38	0.69±0.16	0.85±0.10
	P value	_	0.7167	0.5582	0.4505	0.3921	0.3650	0.7654
Norepinephrine	***Media***	1.00±0.07	0.98±0.13	1.03±0.08	0.93±0.11	0.80±0.12	0.75±0.06	0.82±0.14
	P value#				0.0001***			
	Cell	ND	ND	ND	ND	ND	ND	ND
	P value	_	_	_	_	_	_	_
Epinephrine	***Media***	1.00±0.14	0.98±0.09	0.98±0.14	0.96±0.13	1.02±0.15	0.97±0.16	1.01±0.14
	P value#				0.9995			
	Cell	1.00±1.73	4.59±1.78	3.34±3.21	1.76±1.54	2.66±2.83	2.33±1.92	0.77±0.67
	P value	_	0.0703	0.4434	0.6012	0.7394	0.6798	0.5103
Tyramine	***Media***	1.00±0.57	0.65±0.25	0.70±0.06	0.59±0.08	0.68±0.21	0.50±0.08	0.65±0.15
	P value#				0.3412			

	Cell	1.00±0.62	0.87±0.27	1.13±0.25	1.16±0.85	1.29±0.90	1.27±0.72	1.36±0.43
	P value	_	0.9606	0.5828	0.7851	0.8347	0.6839	0.4301
3-Methoxy-tyramine	***Media***	1.00±0.10	1.46±0.34	2.24±0.13	2.75±1.18	3.53±1.35	4.00±1.02	4.16±1.52
	P value#				1.9187E-07***			
	Cell	1.00±0.62	1.88±0.87	2.29±1.45	2.37±2.36	2.04±1.54	2.35±1.03	2.34±0.73
	P value	_	0.2416	0.1621	0.3613	0.3344	0.0890	0.0749
Tryptophan	***Media***	1.00±0.01	0.82±0.12	0.95±0.07	0.80±0.33	0.66±0.17	0.49±0.26	0.82±0.44
	P value#				0.0023**			
	Cell	1.00±0.25	1.23±0.84	1.64±0.65	1.42±0.49	0.87±1.06	0.77±0.12	1.84±1.09
	P value	_	0.8371	0.1551	0.3033	0.4284	0.2425	0.2156
Serotonin	***Media***	1.00±0.11	0.90±0.11	0.92±0.03	0.90±0.07	0.94±0.05	0.89±0.08	0.97±0.11
	P value#				0.5189			
	Cell	1.00±0.55	0.37±0.11	0.32±0.12	0.22±0.06	0.40±0.15	0.43±0.09	0.62±0.07
	P value	_	0.1040	0.0806	0.0307*	0.1332	0.1561	0.4330
Melatonin	***Media***	1.00±0.10	0.90±0.13	0.95±0.13	0.83±0.10	0.86±0.01	0.85±0.04	0.91±0.04
	P value#				0.2287			
	Cell	1.00±0.78	0.49±0.43	0.62±0.22	0.39±0.09	0.05±0.08	0.18±0.31	0.17±0.21
	P value	_	0.9845	0.5372	0.1711	0.5957	0.9876	0.3338
Glutamate	***Media***	1.00±0.08	1.03±0.10	1.14±0.11	1.13±0.23	1.19±0.16	1.20±0.20	1.32±0.40
	P value#				0.4786			
	Cell	1.00±0.45	0.95±0.59	0.73±0.30	0.78±0.54	0.57±0.53	0.66±0.24	0.82±0.30
	P value	_	0.7747	0.4285	0.5202	0.2646	0.2781	0.6220
Acetylcholine	***Media***	1.00±0.08	0.96±0.12	1.06±0.05	1.04±0.03	1.05±0.14	1.11±0.03	1.07±0.05
	P value#				0.3941			
	Cell	1.00±0.46	0.53±0.14	0.95±0.58	1.05±0.78	0.66±0.26	0.87±0.49	0.54±0.18
	P value	_	0.1252	0.8270	0.8835	0.3307	0.6971	0.1568
Adenosine	***Media***	1.00±0.16	1.06±0.08	1.19±0.22	1.09±0.15	1.08±0.06	1.11±0.09	1.00±0.09

	P value#				0.7904			
	Cell	1.00±0.73	2.31±1.95	2.89±2.26	3.02±1.86	3.81±2.19	5.23±1.34	6.90±1.84
	P value	_	0.4738	0.3365	0.1807	0.0935	0.0215*	0.0141*
Histamine	***Media***	1.00±0.03	0.99±0.11	1.02±0.15	1.01±0.13	1.03±0.10	1.02±0.14	1.05±0.13
	P value#				0.9533			
	Cell	1.00±1.16	0.74±0.31	0.34±0.35	0.82±0.92	1.19±1.14	1.11±0.91	1.37±0.56
	P value	_	0.2654	0.2378	0.3398	0.6693	0.6387	0.8621

1. The " p value# "was derived using repeated measures ANOVA.

2. The" p-value" was calculated after applying a logarithmic transformation, followed by a Student's t-test.

3. *: p<0.05, **: p<0.01, ***: p<0.001

Supplemental Table S3. The neurotransmitter changes in PC12 upon rotenone pretreatment and NGF stimulation. The concentrations of neurotransmitters were normalized against the neurotransmitter concentrations of the control without differentiation.

Compounds	*Ratio±SD*	*Diff.1*	*Diff.4*	*Diff.7*
Phenylalanine	***Control***	0.31±0.09	0.54±0.27	0.90±0.29
	Rotenone	0.48±0.09<br>p = 0.1046	1.39±0.33<br>p = 0.0551	1.11±0.21<br>p = 0.3673
Tyrosine	***Control***	0.42±0.24	0.61±0.40	0.74±0.25
	Rotenone	1.14±0.60<br>p = 0.0820	2.05±0.51<br>p = 0.0661	1.04±0.23<br>p = 0.2084
Dopamine	***Control***	0.75±0.31	0.54±0.26	0.64±0.08
	Rotenone	0.60±0.20<br>p = 0.6102	0.23±0.05<br>p = 0.0927	0.48±0.08<br>p = 0.0678
Epinephrine	***Control***	0.09±0.16	0.16±0.14	0.07±0.06
	Rotenone	3.74±0.82<br>p = 0.0179*	1.44±0.37<br>p = 0.0629	1.42±0.20<br>p = 0.0707
Tyramine	***Control***	0.66±0.41	0.77±0.56	0.90±0.28
	Rotenone	0.74±0.74<br>p = 0.2926	1.09±0.54<br>p = 0.4976	0.73±0.51<br>p = 0.5020
3-Methoxy-tyramine	***Control***	0.75±0.46	1.77±1.76	1.74±0.54
	Rotenone	0.31±0.19<br>p = 0.1361	0.20±0.06<br>p = 0.0293*	0.61±0.14<br>p = 0.0128*
Tryptophan	***Control***	0.48±0.12	0.69±0.24	0.89±0.53
	Rotenone	2.88±0.73<br>p = 0.0010**	2.26±0.76<br>p = 0.0202*	1.17±0.75<br>p = 0.6205
Serotonin	***Control***	1.14±0.63	0.25±0.06	0.71±0.08
	Rotenone	4.09±0.93<br>p = 0.0275*	1.51±0.32<br>p = 0.0010**	1.59±0.35<br>p = 0.0058**
Melatonin	***Control***	1.18±0.92	0.46±0.11	0.20±0.25
	Rotenone	3.91±0.78<br>p = 0.0365*	1.46±0.16<br>p = 0.0012**	1.61±0.33<br>p = 0.0896
Glutamate	***Control***	0.45±0.20	0.35±0.24	0.37±0.14
	Rotenone	0.84±0.31<br>p = 0.1221	0.46±0.07<br>p = 0.4219	0.49±0.06<br>p = 0.2542
Acetylcholine	***Control***	1.16±0.53	1.22±0.91	0.63±0.21
	Rotenone	4.18±0.56	1.45±0.30	1.59±0.09

		p = 0.0085**	p = 0.4940	p = 0.0090**
Adenosine	***Control***	0.33±0.24	0.99±0.61	2.26±0.60
	Rotenone	0.39±0.02	0.77±0.08	1.09±0.38
		p = 0.4608	p = 0.9245	p = 0.0394*
Histamine	***Control***	1.24±1.44	1.02±1.14	1.69±0.69
	Rotenone	4.50±0.59	1.53±0.42	1.47±0.25
		p = 0.0326*	p = 0.2992	p = 0.7741

: p<0.05, **: p<0.01, ***: p<0.001

Supplemental Table S4. The neurotransmitter changes after the cells be treated with 10 nM, 20 nM and 100 nM rotenone for 48 hours. The concentrations of neurotransmitters were normalized to the neurotransmitter concentrations on non-differentiated control. The p-values are the results from t-test statistical analysis against non-differentiated control.

Compounds	***Ratio±SD***	***Nondiff ctrl***	***Nondiff rot***	***Diff.8 ctrl***	***10 nM rot***	***20 nM rot***	***100 nM rot***
Phenylalanine	***Media***	1.00±0.04	0.98±0.03	1.34±0.03	1.39±0.03	1.40±0.01	0.79±0.00
	cell	1.00±0.23	1.26±0.26	0.54±0.08	0.09±0.04	0.07±0.01	0.10±0.02
Tyrosine	***Media***	1.00±0.05	1.05±0.07	1.46±0.18	1.99±0.10	1.88±0.17	0.84±0.04
	cell	1.00±0.32	1.35±0.30	0.62±0.10	0.16±0.10	0.10±0.03	0.09±0.01
L-DOPA	***Media***	ND	ND	1.00±1.00	5.64±0.70	7.37±2.53	ND
	cell	ND	ND	ND	ND	ND	ND
Dopamine	***Media***	ND	ND	1.00±1.23	13.19±2.46	14.76±0.70	1.95±0.33
	cell	1.00±0.12	1.15±0.19	0.77±0.21	0.02±0.02	0.01±0.01	0.04±0.03
Epinephrine	***Media***	1.00±0.54	1.32±0.37	ND	ND	ND	ND
	cell	1.00±0.19	3.10±0.76	ND	ND	ND	ND
Tyramine	***Media***	1.00±0.44	1.53±0.32	ND	ND	ND	1.28±0.23
	cell	1.00±0.12	1.52±0.80	1.51±0.85	ND	ND	0.59±0.29
3-Methoxytyramine	***Media***	1.00±0.26	0.79±0.23	38.83±1.80	52.26±6.18	52.50±6.99	20.71±2.88
	cell	1.00±0.19	0.89±0.71	0.84±0.26	0.23±0.20	ND	ND
Tryptophan	***Media***	1.00±0.10	0.99±0.10	1.68±0.07	1.87±0.22	1.74±0.21	0.81±0.13
	cell	1.00±0.46	0.82±0.15	0.37±0.04	0.08±0.05	0.11±0.05	0.11±0.13
Serotonin	***Media***	ND	ND	ND	ND	ND	ND
	cell	1.00±0.35	2.85±0.88	0.67±0.09	ND	ND	ND
5-HIAA	***Media***	ND	0.95±0.32	ND	ND	ND	0.52±0.22
	cell	1.00±1.15	2.76±0.27	1.44±0.63	ND	ND	1.23±0.53
Glutamate	***Media***	1.00±0.16	1.08±0.04	2.03±0.14	1.59±0.13	1.61±0.08	0.88±0.17
	cell	1.00±0.02	1.00±0.34	0.29±0.08	0.01±0.01	0.03±0.03	0.02±0.01

GABA	***Media***	1.00±0.56	1.41±0.33	0.79±0.26	0.67±0.23	0.42±0.10	0.70±0.27
	cell	1.00±1.05	1.60±0.61	0.68±0.25	1.29±0.41	1.00±0.93	0.99±0.43
Acetylcholine	***Media***	1.00±0.51	1.05±0.32	0.43±0.03	0.48±0.24	0.70±0.29	0.94±0.36
	cell	1.00±1.01	2.08±0.83	0.08±0.02	0.05±0.01	ND	ND
Adenosine	***Media***	1.00±0.17	0.97±0.21	0.73±0.04	0.76±0.09	0.60±0.08	0.66±0.07
	cell	1.00±0.27	1.13±0.17	5.03±1.28	0.22±0.06	0.18±0.03	0.32±0.13

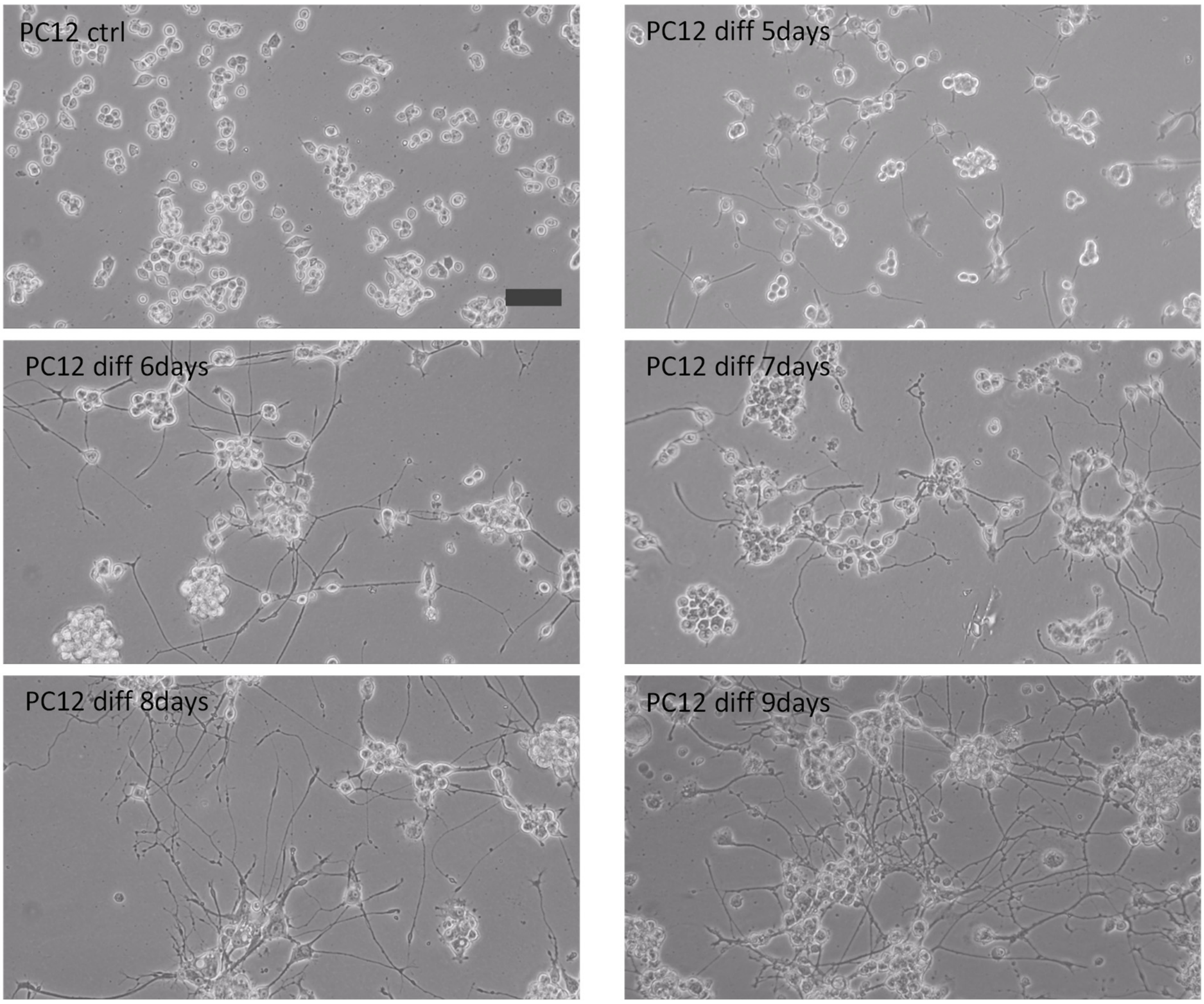

Supplemental Figure S1. The morphological change of the differentiation of PC12 cells. Pheochromocytoma cells(PC12) were stimulated by 50 nM of neural growth factor(NGF). The scale bar is 50 μm.

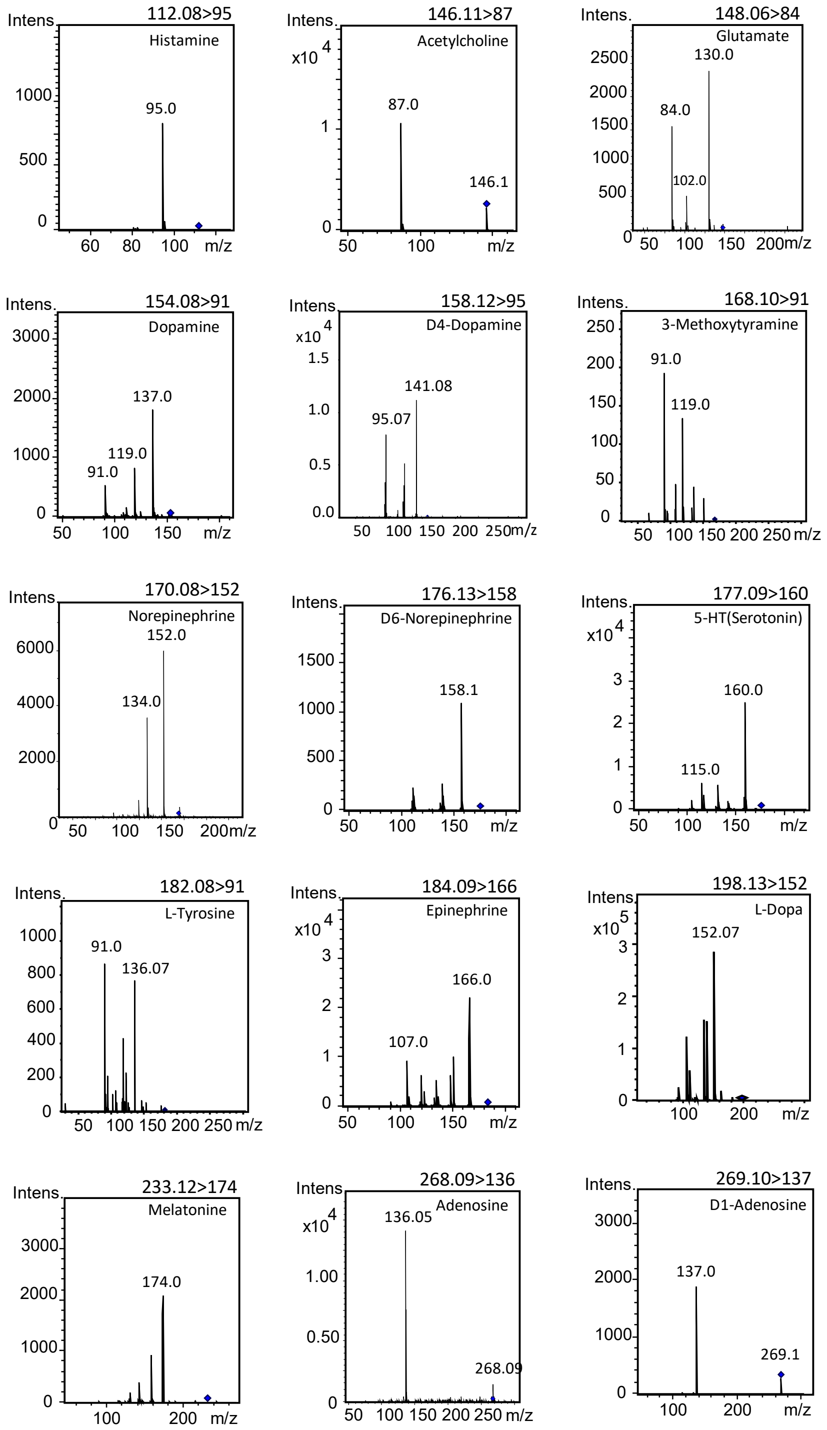

Supplemental Figure S2. Fragmentation pattern of the neurotransmitter standards. The MS/MS spectra of neurotransmitter standards from Q-TOF. The number above each spectrum were the selected precursor ion and product ion for quantification. The blue rhombus indicated the precursor ions of the neurotransmitter standards.

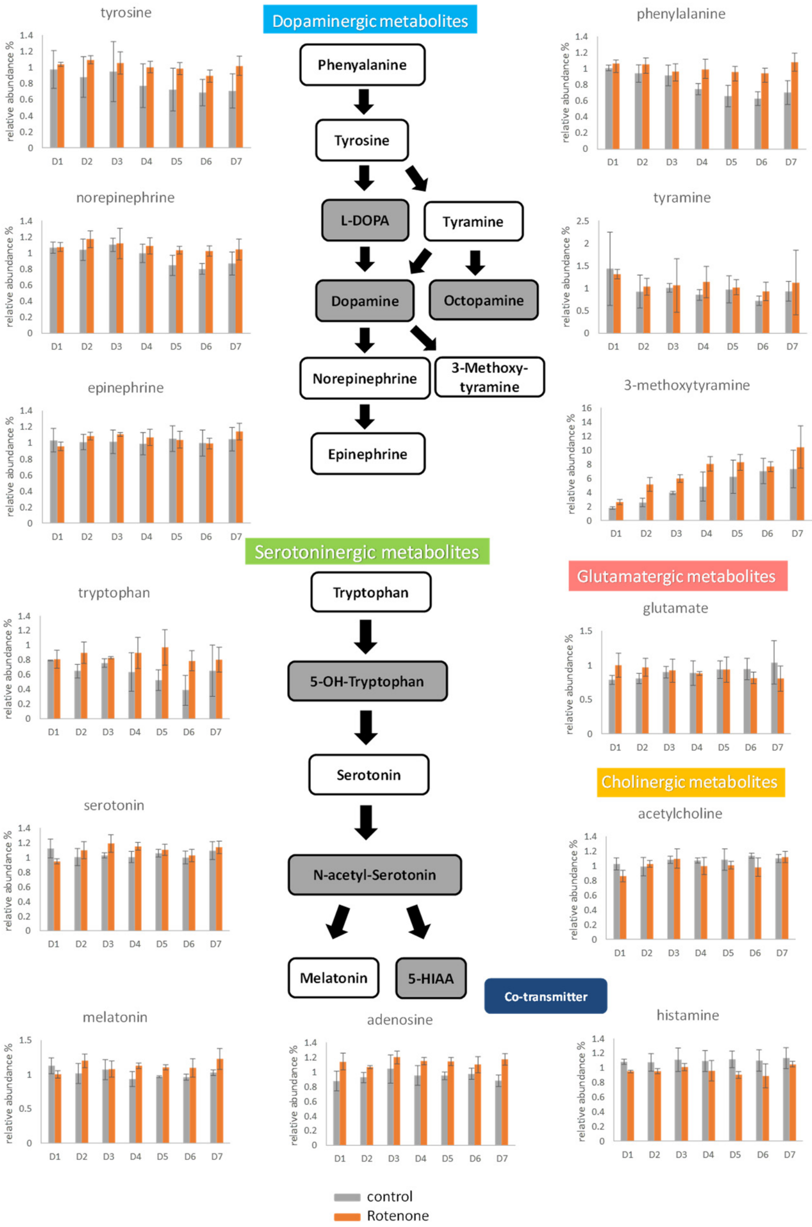

Supplemental Figure 3. The release of neurotransmitter by PC12 upon rotenone pretreatment and NGF stimulation PC12 was pre-treated with 100 nM rotenone and then the differentiation was stimulated by NGF for 7 days. The neurotransmitters were quantitated by Q-TOF mass spectrometry.
